# Long-Term Burden of Adolescent-Onset Endometriosis: A Case Report Highlighting Recurrent Disease and Fertility-Preserving Dilemmas

**DOI:** 10.7759/cureus.97388

**Published:** 2025-11-20

**Authors:** Airi Kuruma, Mina Sakata, Erika Nakatsuka, Mahiru Kawano, Michiko Kodama

**Affiliations:** 1 Department of Obstetrics and Gynecology, Graduate School of Medicine, University of Osaka, Suita, JPN; 2 Department of Obstetrics and Gynecology, Rinku General Medical Center, Izumisano, JPN

**Keywords:** adolescent endometriosis, chronic pelvic pain, fertility preservation, infertility, recurrence

## Abstract

Adolescent-onset endometriosis is increasingly recognized as a distinct clinical challenge. It often requires years to achieve a diagnosis, leading to prolonged physical and psychological distress and significantly impairing quality of life. We report the case of a 37-year-old woman with endometriosis first diagnosed in adolescence. Long‑term management was complicated by an inability to tolerate continuous hormonal therapy because of adverse effects, resulting in multiple recurrences. After infertility treatment, she achieved pregnancy but required an emergency cesarean section for placenta previa. Due to the persistent severe treatment-refractory pain, she underwent a laparoscopic total hysterectomy with bilateral adnexectomy. Postoperatively, pain resolved completely, and she has remained symptom-free on hormone replacement therapy. This case illustrates the substantial lifelong burden that adolescent‑onset endometriosis can impose, including recurrent disease, complex fertility decisions, and significant impacts on quality of life. Early recognition and sustained multidisciplinary follow‑up, considering fertility goals, are essential to improve long‑term outcomes.

## Introduction

Endometriosis is a disease in which an endometrium-like tissue grows outside the uterus, associated with adverse obstetric outcomes, chronic pelvic pain, infertility, and an increased risk of cancer. Endometriosis affects approximately 10% of women of reproductive age and is recognized as an estrogen-dependent chronic inflammatory disease [1]. Recently, endometriosis has been reported to be found in 60% to 70% of adolescents having dysmenorrhea or chronic pelvic pain [2]. In adolescence, it may present with severe dysmenorrhea, chronic pelvic pain, or atypical symptoms such as back pain and gastrointestinal complaints [3]. Adolescent-onset endometriosis tends to follow a prolonged clinical course and may result in infertility, chronic pain, and repeated surgeries. Moreover, recent studies have reported associations with adverse obstetric outcomes [4-6]. Some cases develop chronic pelvic pain, including neuropathic, nociceptive, and nociplastic pain [7]. Therefore, early diagnosis and continuous management that take into account the patient’s overall life plan are essential to prevent progression. Here, we report a case of adolescent-onset endometriosis with poor adherence to hormonal therapy, resulting in recurrent disease, multiple surgeries, severe neurological symptoms, and, ultimately, hysterectomy despite fertility desire.

## Case presentation

**Figure 1:**
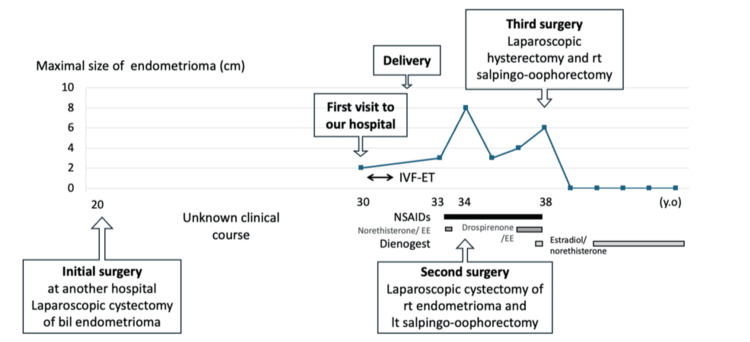
Summary of the clinical course. IVF-ET = in vitro fertilization and embryo transfer; NSAID = nonsteroidal anti-inflammatory drug

We report the case of a woman with adolescent-onset endometriosis who underwent laparoscopic bilateral cystectomy of endometriotic cysts at age 20 at another hospital. She first visited our hospital at age 29. Until then, details of the previous surgery and the following clinical course were unclear. According to her, recurrent pain had been managed mainly with nonsteroidal anti-inflammatory drugs (NSAIDs), and dienogest was discontinued due to depressive symptoms. Her clinical course is summarized in Figure 1.

She was referred to another clinic to undergo in vitro fertilization and embryo transfer (IVF-ET) and conceived in the fourth cycle. She delivered at 33 weeks and 6 days by emergency cesarean section due to placenta previa with hemorrhage at age 31 at our hospital. Intraoperative findings included complete obliteration of the pouch of Douglas; estimated blood loss was 2,200 mL.

**Figure 2:**
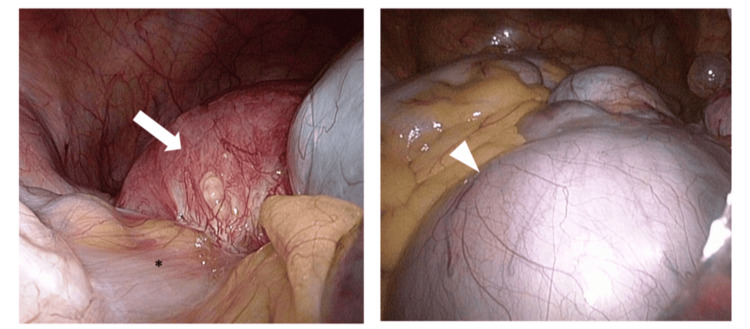
Intraoperative images during the second surgery. (Left) Dense adhesions existed between posterior uterine wall and sigmoid colon, extending to complete obliteration of the pouch of Douglas. White arrow: uterus, asterisk: sigmoid colon (Right) White triangle indicates an 8-cm right ovarian endometrioma.

Following delivery, she resumed spontaneous menses and had mild cyst enlargement. A combined oral contraceptive (COC), containing 35 µg ethinylestradiol and 1 mg norethisterone, was restarted but discontinued due to childcare demands. Recurrent low back pain was managed at a local pain clinic with NSAIDs and nerve blocks. At age 34, imaging detected an 8‑cm right ovarian endometriotic cyst. She underwent laparoscopic right ovarian cystectomy, bilateral salpingectomy, and adhesiolysis. The revised American Society for Reproductive Medicine (rASRM) score was 130 (Stage IV) (Figure 2). Postoperative pain improved substantially. Postoperative hormone suppressive therapy was not administered as the patient wished to conceive. Subsequently, due to the enlargement of the endometriotic cyst, continuous COC (containing 20 μg ethinyl estradiol and 3 mg drospirenone) was administered for one year.

**Figure 3:**
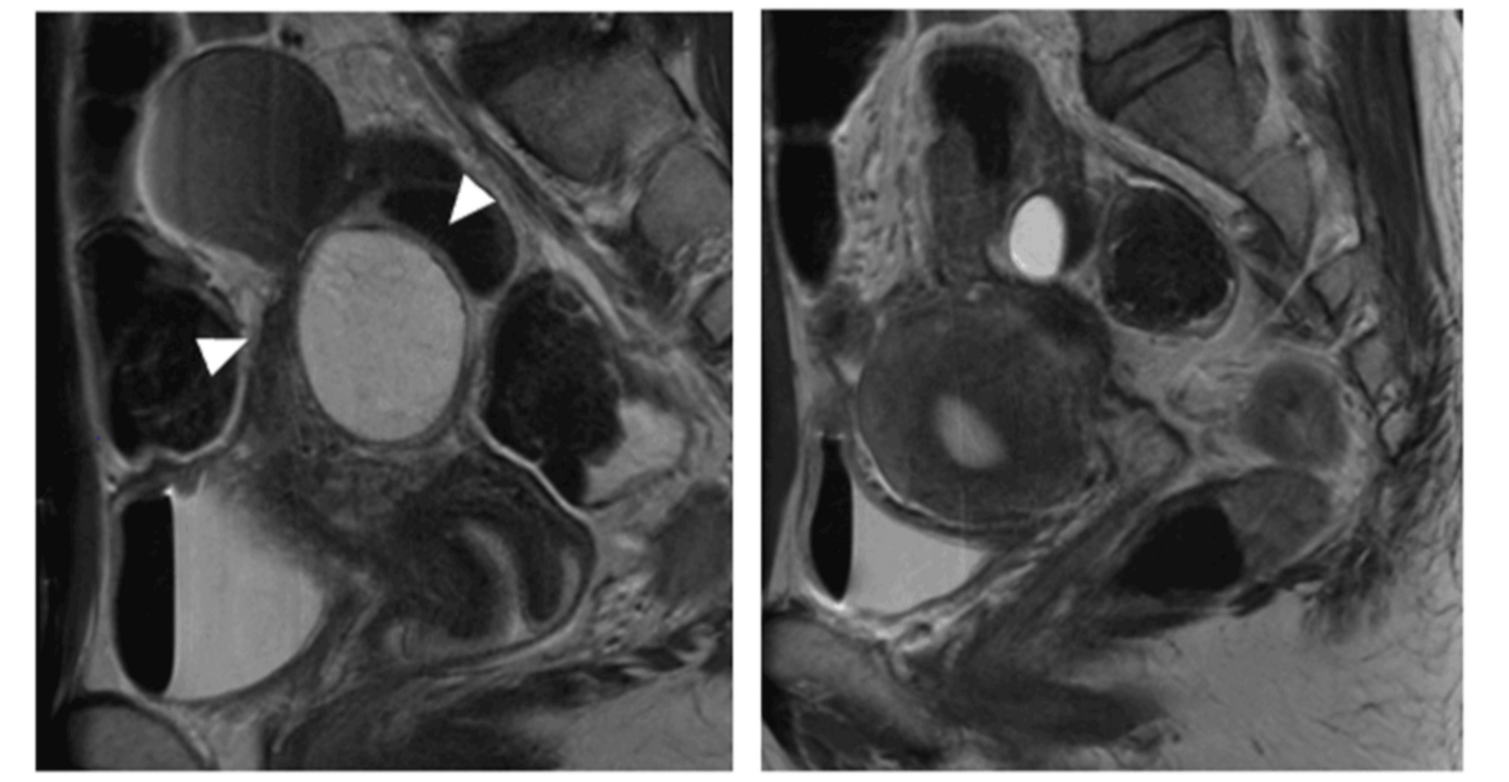
MRI before the third surgery. (Left) Right endometrioma is indicated by white triangles. (Right) Uterine axis was displaced to the left.

During this treatment, she developed severe cyclical pain radiating from the right buttock to the right lower limb with difficulty standing and walking, and presented emergently at age 38. Orthopedic evaluation documented right L5 radiculopathy. Pelvic examination revealed a firm 1‑cm nodule in the right pouch of Douglas; palpation reproduced electric shock-like pain radiating to the right leg. Pelvic MRI showed bilateral ovarian endometriomas and dense adhesions involving the ovaries, uterus, and rectum, without clear deep infiltrative endometriosis encasement of the lumbosacral plexus or sciatic nerve (Figure 3).

**Figure 4:**
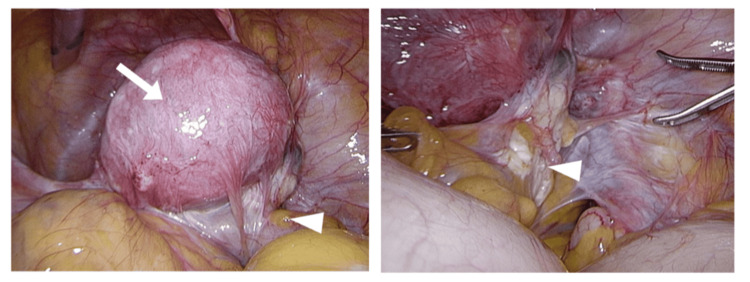
Intraoperative images during the third surgery. (Left) Extensive severe adhesion existed around the posterior uterine wall, sigmoid colon, and right endometrioma. White arrow indicates uterus, and white triangle indicates right endometrioma. (Right) Due to the adhesions around the right ovary, the pouch of Douglas could not be observed.

Conservative management, including escalation of analgesics, repeat nerve blocks, and reinstitution of dienogest (2 mg/day), yielded only transient relief; opioids failed to achieve acceptable pain control. Seventeen years had passed since the initial diagnosis of endometriosis. After multidisciplinary counseling on fertility implications and surgical risks, she elected definitive surgery. Laparoscopic simple hysterectomy with bilateral adnexectomy and adhesiolysis was performed; intraoperative rASRM score was 112 (Stage IV) (Figure 4). By postoperative day one, the radicular pain had resolved, and ambulation returned; analgesia was maintained with NSAIDs alone. During recent follow‑up, she has remained pain‑free and off analgesics, with no recurrence of endometriosis detected during pelvic examination and ultrasonography for seven years and nine months following the definitive procedure. Hormone replacement therapy, estradiol and norethisterone, has been administered for vasomotor and menopausal symptoms.

## Discussion

Adolescent-onset endometriosis significantly affects both physical and psychological health [8]. Laparoscopically confirmed endometriosis has been reported in 62% of adolescents undergoing diagnostic laparoscopy, 75% of those with treatment-resistant chronic pelvic pain, and 70% of those with dysmenorrhea [9]. Greene et al. found an average delay of six years from symptom onset to the first consultation, followed by more than five additional years to definitive diagnosis, during which symptoms may progress [10]. Diagnostic delay reflects patient-related factors, including limited awareness of dysmenorrhea, delayed health-seeking, and reluctance to initiate hormonal therapy, as well as clinician factors, including the cultural perception of dysmenorrhea as “normal” and presentation with atypical gastrointestinal or musculoskeletal complaints that lead to underdiagnosis [2,11]. Even in adolescents, ovarian endometriomas and deep infiltrating endometriosis can occur, and symptom severity does not always correlate with disease extent, increasing the risk of underestimation [12]. Management must be comprehensive and address schooling, employment, partnership, fertility, and parenting. Long-term medical therapy with COCs or progestins is the mainstay and should be individualized according to side effects, lifestyle, and contraindications [13]. Surgery is often employed for diagnosis or pain relief [13], but postoperative recurrence is common; reported recurrence rates vary widely (6-67%) depending on definitions and follow-up duration. Higher recurrence is associated with more advanced stage disease and younger age [14]. Repeat surgeries are linked to longer operative times, extensive adhesiolysis, and increased complication rates [15].

Patients with endometriosis face multifaceted challenges, including physical, reproductive, and psychological issues. For those desiring pregnancy, dyspareunia and infertility are major concerns [16]. Moreover, endometriosis is associated with adverse obstetric outcomes such as spontaneous hemoperitoneum in pregnancy, placenta previa, preterm birth, higher cesarean delivery rates, and increased intrapartum blood loss [4-6].

Regarding fertility, the European Society of Human Reproduction and Embryology guideline states that surgery may be considered for Stage I-II disease to improve natural conception rates. Still, routine pre-assisted reproductive technology surgery is not recommended, as benefits are uncertain and ovarian reserve may be compromised [13].

Endometriosis-associated chronic pelvic pain is a significant clinical problem characterized by heterogeneous symptoms and potentially comorbid with conditions possibly linked through central and peripheral sensitization, such as irritable bowel syndrome, interstitial cystitis/painful bladder syndrome, migraine, and fibromyalgia [7,17]. Endometriosis can cause several types of neurological pains, including neuropathic, nociceptive, and nociplastic pain [7]. The chronic pain in this case might be nociceptive, because it was quickly resolved by the removal of endometriotic disease and uterosacral ligament resection during hysterectomy without extending into the sciatic nerve area. Nociceptive pain arising from central sensitization is often reported to be difficult to manage, and neuropathic pain can be exacerbated by direct nerve damage. Additionally, women with endometriosis have an increased risk of ovarian, thyroid, and breast cancers, warranting counseling on general cancer-prevention measures [18].

This case exemplifies the complexities of adolescent-onset endometriosis. Despite the patient’s desire to preserve fertility, multiple surgeries, including a hysterectomy, were required due to intolerance to long-term hormonal therapy and the nature of the endometriosis, which makes complete surgical resection difficult.

## Conclusions

Adolescent endometriosis is a chronic, estrogen-dependent condition that can profoundly affect long-term quality of life and reproductive outcomes. Early diagnosis and sustained medical therapy alongside a shared decision-making process may help minimize disease progression and preserve fertility while reducing the need for repeated surgeries.

## References

[1] Zondervan KT, Becker CM, Missmer SA (2020). Endometriosis. N Engl J Med.

[2] Dun EC, Kho KA, Morozov VV, Kearney S, Zurawin JL, Nezhat CH (2015). Endometriosis in adolescents. JSLS.

[3] Beloshevski B, Shimshy-Kramer M, Yekutiel M, Levinsohn-Tavor O, Eisenberg N, Smorgick N (2024). Delayed diagnosis and treatment of adolescents and young women with suspected endometriosis. J Gynecol Obstet Hum Reprod.

[4] Busnelli A, Di Simone N, Somigliana E (2024). Untangling the independent effect of endometriosis, adenomyosis, and ART-related factors on maternal, placental, fetal, and neonatal adverse outcomes: results from a systematic review and meta-analysis. Hum Reprod Update.

[5] Matsuzaki S, Nagase Y, Ueda Y (2021). The association of endometriosis with placenta previa and postpartum hemorrhage: a systematic review and meta-analysis. Am J Obstet Gynecol MFM.

[6] Lier MC, Malik RF, Ket JC, Lambalk CB, Brosens IA, Mijatovic V (2017). Spontaneous hemoperitoneum in pregnancy (SHiP) and endometriosis - a systematic review of the recent literature. Eur J Obstet Gynecol Reprod Biol.

[7] Karp BI, Stratton P (2023). Endometriosis-associated chronic pelvic pain. Med.

[8] Gallagher JS, DiVasta AD, Vitonis AF, Sarda V, Laufer MR, Missmer SA (2018). The impact of endometriosis on quality of life in adolescents. J Adolesc Health.

[9] Janssen EB, Rijkers AC, Hoppenbrouwers K, Meuleman C, D'Hooghe TM (2013). Prevalence of endometriosis diagnosed by laparoscopy in adolescents with dysmenorrhea or chronic pelvic pain: a systematic review. Hum Reprod Update.

[10] Greene R, Stratton P, Cleary SD, Ballweg ML, Sinaii N (2009). Diagnostic experience among 4,334 women reporting surgically diagnosed endometriosis. Fertil Steril.

[11] Simpson CN, Lomiguen CM, Chin J (2021). Combating diagnostic delay of endometriosis in adolescents via educational awareness: a systematic review. Cureus.

[12] Millischer AE, Santulli P, Da Costa S, Bordonne C, Cazaubon E, Marcellin L, Chapron C (2023). Adolescent endometriosis: prevalence increases with age on magnetic resonance imaging scan. Fertil Steril.

[13] Becker CM, Bokor A, Heikinheimo O (2022). ESHRE guideline: endometriosis. Hum Reprod Open.

[14] Selçuk I, Bozdağ G (2013). Recurrence of endometriosis; risk factors, mechanisms and biomarkers; review of the literature. J Turk Ger Gynecol Assoc.

[15] Tummers FH, Peltenburg SI, Metzemaekers J, Jansen FW, Blikkendaal MD (2023). Evaluation of the effect of previous endometriosis surgery on clinical and surgical outcomes of subsequent endometriosis surgery. Arch Gynecol Obstet.

[16] Vercellini P, Viganò P, Bandini V, Buggio L, Berlanda N, Somigliana E (2023). Association of endometriosis and adenomyosis with pregnancy and infertility. Fertil Steril.

[17] Song SY, Jung YW, Shin W (2023). Endometriosis-related chronic pelvic pain. Biomedicines.

[18] Kvaskoff M, Mahamat-Saleh Y, Farland LV (2021). Endometriosis and cancer: a systematic review and meta-analysis. Hum Reprod Update.

